# Data-driven assessment of dimension reduction quality for single-cell omics data

**DOI:** 10.1016/j.patter.2022.100465

**Published:** 2022-03-11

**Authors:** Xiaoru Dong, Rhonda Bacher

**Affiliations:** 1 Department of Biostatistics, University of Florida, Gainesville, FL, USA

## Abstract

Dimension reduction (DR) techniques have become synonymous with single-cell omics data due to their ability to generate attractive visualizations and enable analyses of high-dimensional data. In this issue of *Patterns*, Johnsona et al. develop a statistical approach to assist in selecting high-quality reduced representations to improve analyses and biological interpretations.

## Main text

Single-cell RNA sequencing (scRNA-seq) experiments have revolutionalized the field of genomics by capturing gene-expression data at the level of individual cells and allowing researchers to uncover biological properties of individual cells in complex tissues. In order to enable such powerful insights, this high-dimensional sequencing data requires a combination of specific preprocessing steps, including quality control, normalization, dimensionality reduction (DR), and clustering.^1^ Following these steps, one has the ability to identify rare cell types, discover trajectories representing biological processes, and identify differentially expressed genes across conditions for particular cell types. However, it has been demonstrated that these analyses and their biological conclusions are influenced by different approaches used for preprocessing.^2^ Notably, DR has been a focus in single-cell analysis given its ubiquitousness among visualization and computational methods.

In general, DR involves projecting high-dimensional data into a lower dimensional space in order to reduce noise signals in the data while retaining key features. The traditional and most familiar form of DR is done via principal component analysis (PCA), which performs linear transformations and preserves the Euclidean distance between features. More recent nonlinear approaches, such as t-distributed stochastic neighbor embedding (t-SNE)^3^ and uniform approximation and projection method (UMAP),^4^ have become popular in single-cell data and are highly regarded for their ability to produce appealing visualizations of cell clusters. This is because they aim to preserve the local structure of the data while typically ignoring or placing less emphasis on the global structure of the data, i.e., the distance between cells. The non-linear algorithms are also stochastic and heavily dependent on hyperparameters chosen by users.^5^ Besides visualization, DR is additionally required for the majority of downstream analyses. Thus, choosing an appropriate DR method, one that is able to retain the structure of original data and impose the least distortion of biological signals, is a priority.

The concern around DR approaches used on single-cell data has largely resulted in developing novel DR methods or heuristic guidelines based on benchmarking studies.^6^^,^^7^ However, choosing an optimal DR method for a given dataset and analysis remains an open question. In this issue of *Patterns*, Johnsona et al.^8^ tackle this problem by developing a quantitative quality assessment scheme: empirical marginal resampling better evaluates dimensionality reduction (EMBEDR). EMBEDR distinguishes those structures in the reduced dimension embedding consistent with those in the original high-dimensional data versus those attributable to noise, allowing users to determine which DR representation captures the structure of the original data most accurately.

The key to EMBEDR’s evaluation is the introduction of a quality statistic termed the empirical embedding statistic, which compares cell-to-cell distance distributions between the original data and its reduced dimension embedding.^8^ The quality statistic is generated for each DR method and compared to the distribution of quality statistics calculated on null datasets generated via marginal resampling. An empirical hypothesis test is performed comparing the sample cell’s quality to the null quality distribution, with p values calculated as the probability that the observed data yield a lower-quality embedding compared to the null datasets. If the p value for a cell is small, it indicates the structure of the cell in the embedding is close to the structure in the original high-dimensional data.

EMBEDR is implemented in Python and provides users multiple evaluations of the DR approaches. For example, visualizing the cell-specific p values provides users a measure of where signals are best preserved in a given embedding and most likely to reflect biological signal. EMBEDR can also be used to select the optimal hyperparameters for a given approach and compare embeddings across DR methods. EMBEDR also allows users to explore the locally optimal embedding for each cell type. Johnson et al. emphasize that the globally optimal embedding does not necessarily mean that the quality in each local cell type is ideal and that performing local optimization may facilitate identification of rare cell types.^8^

As interest in scRNA-seq technologies grows, datasets are increasing in size and complexity. DR will continue to be a key step for visualizing and analyzing single-cell RNA data, and identifying an optimal DR method remains a high priority. Johnson et al. demonstrate EMBEDR’s ability to assist users in selecting the most appropriate DR method objectively by quantitatively measuring each cell’s quality in embeddings. Given the increasing number of methodologies that are being developed for single-cell analyses, we anticipate a greater emergence and focus on data-driven methodology selections^9^ and comprehensive evaluation frameworks^10^ in the coming years.
